# Calreticulin enhances gastric cancer metastasis by dimethylating H3K9 in the E-cadherin promoter region mediating by G9a

**DOI:** 10.1038/s41389-022-00405-7

**Published:** 2022-05-31

**Authors:** Lina Wang, Jun Chen, Qianfei Zuo, Chunmei Wu, Ting Yu, Pengfei Zheng, Hui Huang, Jun Deng, Lichao Fang, Huamin Liu, Chenghong Li, Peiwu Yu, Quanming Zou, Junsong Zheng

**Affiliations:** 1 Department of Clinical and Military Laboratory Medicine, College of Medical Laboratory Science, Army Medical University, No. 30 Gaotanyan Street, 400038 Chongqing, China; 2 Department of General Surgery and Center of Minimal Invasive Gastrointestinal Surgery, Southwest Hospital, Army Medical University, No. 30 Gaotanyan Street, 400038 Chongqing, China; 3 National Engineering Research Center of Immunological Products, Department of Microbiology and Biochemical Pharmacy, College of Pharmacy, Army Medical University, No. 30 Gaotanyan Street, 400038 Chongqing, China; 4 Department of medicinal chemistry, College of Pharmacy, Army Medical University, No. 30 Gaotanyan Street, 400038 Chongqing, China

**Keywords:** Oncogenes, Cell biology

## Abstract

The latest study shows that gastric cancer (GC) ranked the fifth most common cancer (5.6%) with over 1 million estimated new cases annually and the fourth most common cause of cancer death (7.7%) globally in 2020. Metastasis is the leading cause of GC treatment failure. Therefore, clarifying the regulatory mechanisms for GC metastatic process is necessary. In the current study, we discovered that calreticulin (CALR) was highly expressed in GC tissues and related to lymph node metastasis and patient’s terrible prognosis. The introduction of CALR dramatically promoted GC cell migration in vitro and in vivo, while the repression of CALR got the opposite effects. Cell migration is a functional consequence of the epithelial-mesenchymal transition (EMT) and is related to adhesion of cells. Additionally, we observed that CALR inhibition or overexpression regulated the expression of EMT markers (E-cadherin, ZO-1, Snail, N-cadherin, and ZEB1) and cellular adhesive moleculars (Fibronectin, integrin β1and MMP2). Mechanistically, our data indicated that CALR could mediate DNA methylation of E-cadherin promoter by interacting with G9a, a major euchromatin methyltransferase responsible for methylation of histone H3 on lysine 9(H3K9me2) and recruiting G9a to the E-cadherin promoter. Knockdown of G9a in CALR overexpressing models restored E-cadherin expression and blocked the stimulatory effects of CALR on GC cell migration. Taken together, these findings not only reveal critical roles of CALR medicated GC metastasis but also provide novel treatment strategies for GC.

## Introduction

Gastric cancer (GC) ranked the fifth most common cancer (5.6%) with over 1 million estimated new cases annually and the fourth most common cause of cancer death (7.7%) globally in 2020 [1, 2]. In 185 countries, the incidence rates of Eastern Asia and Eastern Europe are highest, and mortality rates remain highest in several South Central Asian countries, including Iran, Afghanistan, Turkmenistan, and Kyrgyzstan, especially in men. Although mortality rates for GC slightly decreased in recent years, clinicians expect to see more GC cases in the future due to aging populations and economic development. In virtue of its frequently advanced stage at diagnosis, high molecular and phenotypical heterogeneity and metastasis as the main causes of GC death, efforts to clarify the molecular mechanisms of GC development and progression and find the potential early detection markers contribute to the precise treatment of GC patients and prolong the overall survival rate.

Calreticulin (CALR), also called CRT, locates in the p13.2–p13.3 region of human chromosome 19 and is a highly conserved chaperone protein that resides primarily in the endoplasmic reticulum [3] and is involved in a spectrum of different physiological and pathological roles, among them, cell adhesion. Additionally, it functions in protein folding quality control and maintenance of calcium homeostasis [4]. CALR is also found in the nucleus, implying that it may have a role in transcriptional activities and gene expression regulation [5]. Because of its multifunction, previous studies have shown a contributing role for CALR in various diseases, including neurodegenerative problems, cancers, autoimmune diseases, and wound healing [6–9]. In addition, new roles of CALR as possible diagnostic markers in blood or urine have emerged due to its secretion [10–12]. In gastric cancer, some researchers found that CALR overexpression was associated with GC invasion, metastasis, lymph node metastasis, angiogenesis, and GC patients’ survival [13]. However, the mechanisms that how CALR mediates GC metastasis remain unclarified, and delineating the mechanism and determining the probable interacting genes and signaling pathways contribute to GC precise targeted therapy. Besides, exploring the diagnostic value of serum CALR is also beneficial to discover novel and specific biomarkers for GC.

Metastasis is the leading cause of GC patients’ death, which involves many processes, including cell adhesion, migration, and invasion. Among them, the EMT process, whose hallmark is the absence of E-cadherin, Integrin β1(ITGB1), and Fibronectin (FN1), plays crucial roles [14–16]. Previous studies revealed that CALR enhanced EGF-induced EMT in pancreatic cancer cells via Integrin/EGFR-ERK/MAPK signaling pathway [17] and repressed E-cadherin expression via Snail (SNAI1)/Slug in Madin-Darby canine kidney cells or mouse embryonic stem cells [18, 19].

Intriguingly, in this study, we found that CALR overexpression significantly promoted GC cells migration in vitro and in vivo, while the CALR-mediated effect could be abrogated by blocking G9a (also called EHMT2, euchromatic histone lysine methyltransferase 2) expression in CALR overexpressing model. Mechanistically, we observed that CALR overexpression or suppression significantly regulated the expression of EMT marker (E-cadherin, ZO-1, Snail, N-cadherin and ZEB1) and cellular adhesive molecular (Fibronectin, integrin β1and MMP2). Besides, we demonstrated that CALR interacted with G9a and mediated DNA methylation of E-cadherin promoter, which resulted in suppression of E-cadherin expression. Additionally, we also discovered that serum CALR was better than traditional diagnostic markers for GC detection. Together, our findings expounded the effects of CALR on GC metastasis in detail and provided novel treatment strategies for GC.

## Results

### CALR is highly expressed in GC tissues and correlated with GC lymph node metastasis and poor prognosis

**Fig. 1:**
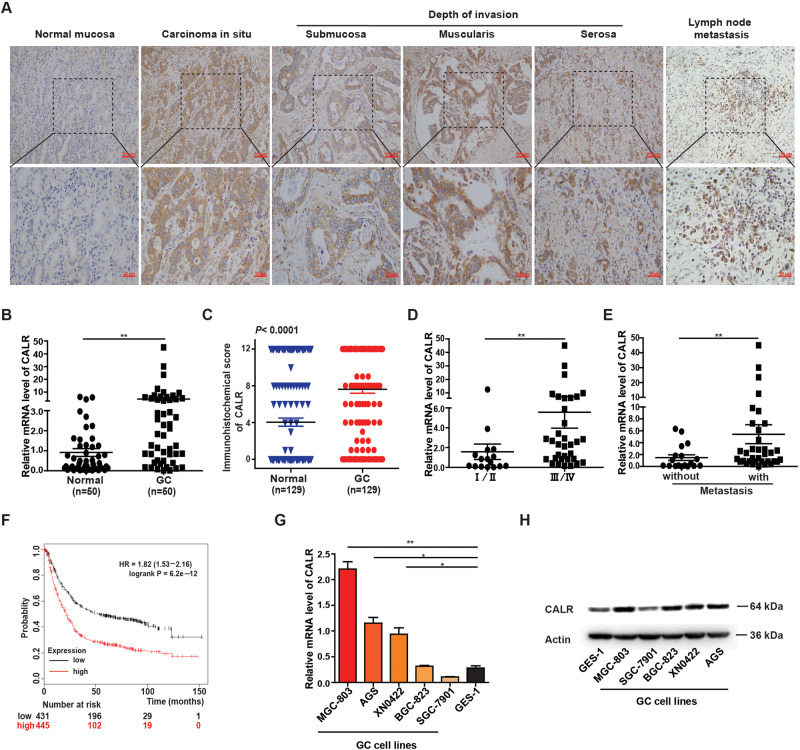
The level and characteristics of CALR in GC tissues or cells. **A** Representative IHC staining of CALR in GC tissues, normal tissues and metastatic lymph node (scale bar = 50 μm). **B** RT-qPCR data for CALR expression in 50 paired fresh GC and normal tissues. **C** Scores analyses of the IHC results; *n* = 129 vs 129. **D**, **E** RT-qPCR data for CALR levels by TNM stage (I + II vs. III + IV) and primary GC tissues with or without lymph node metastasis. **F** Kaplan–Meier analysis of 876 GC patients implyed that high CALR expression was associated with shorter overall survival. **G**, **H** the mRNA and protein level of CALR in normal gastric mucosal epithelial cells (GES-1) and the GC cells (SGC-7901, MGC-803, BGC-823, AGS and XN0422). The data are presented as the mean ± SEM. *P < 0.05, **P < 0.01, ***P < 0.001.

First, our data revealed that CALR protein and mRNA levels were significantly upregulated in GC tissues compared with matched adjacent normal tissues by IHC and qRT-PCR assays. Moreover, the intensity of CALR staining in tumor tissues increased with invasion depth and metastatic lymph node (Fig. 1A–C). These results were consistent with data from the TCGA database (Supplementary Fig. 1A). And then, we analyzed the relationship between CALR expression and the clinical features, and we found that high expression of CALR correlated with GC patients’ TNM stage, distant metastasis, histological differentiation, and tumor size (Fig. 1D, E, Table 1). Furthermore, we also determined that the increased expression of CALR was related to GC grade, postoperative recurrence, and tumor involvement of lymphatic vessels (Supplementary Fig. 1B–E) by analyzing Oncomine data (https://www.oncomine.org/), and overall survival analysis showed a worse survival rate in the GC patients with high CALR expression by Kaplan–Meier Plotter analysis (Fig. 1F) (http://kmplot.com/). These results coincided with Chiung-Nien Chen’s report [13]. Nevertheless, CALR was a secreted protein, so we also examined CALR expression in GC patients’ serum for further exploring its diagnostic values for GC. As a result, receiver operating characteristic (ROC) curve analysis revealed a high AUC value of 0.802 (95% CI, 0.701–0.902) in distinguishing GC from the healthy serums, significantly better than traditional diagnostic markers (CA19-9 and CEA) (Supplementary Fig. 1F). Additionally, we also explored the levels of CALR protein and mRNA in normal gastric mucosal epithelial cells (GES-1) and GC cell lines (SGC-7901, BGC-823, AGS, XN0422, and MGC-803). The results indicated that CALR was highly upregulated in GC cell lines (MGC-803, AGS, and XN0422) and downregulated in SGC-7901 cells compared with GES-1 (Fig. 1G, H). Taken together, the above results demonstrated that CALR expression is significantly increased in GC and is related to GC metastasis. The upregulation signature of CALR expression in GC patients’ serum may be a novel biomarker for the GC diagnosis.

**Table 1:** Relationships between CALR expression (qRT-PCR) in GC tissues and various clinicopathological variables.

Variables	Total	CALR expression			
		Low	High	Mean score ± SD	*P* value
Age					0.263
≥60	47	21	26	7 ± 5.19	
<60	82	21	61	8 ± 4.74	
Gender					0.089
Female	36	11	25	9 ± 4.41	
Male	93	35	58	7 ± 5.04	
Tumor location					0.528
Pylorus and antrum	43	13	30	8 ± 4.91	
Cardia	22	9	13	7 ± 5.36	
Body of fundus	64	22	42	7 ± 4.82	
Tumor stage					0.003**
I–II	27	18	9	5 ± 5.66	
III–IV	102	28	74	8 ± 4.55	
Histological differentiation					0.046*
Low differentiation	83	23	60	8 ± 4.80	
Moderate or well differentiation	46	25	21	6 ± 5.15	
Tumor size (cm)					< 0.0001***
<5	63	35	28	5 ± 4.94	
≥5	66	10	56	9 ± 3.99	

*P < 0.05, **P < 0.01, ***P < 0.001.

### CALR enhances GC cell migration in vitro

**Fig. 2:**
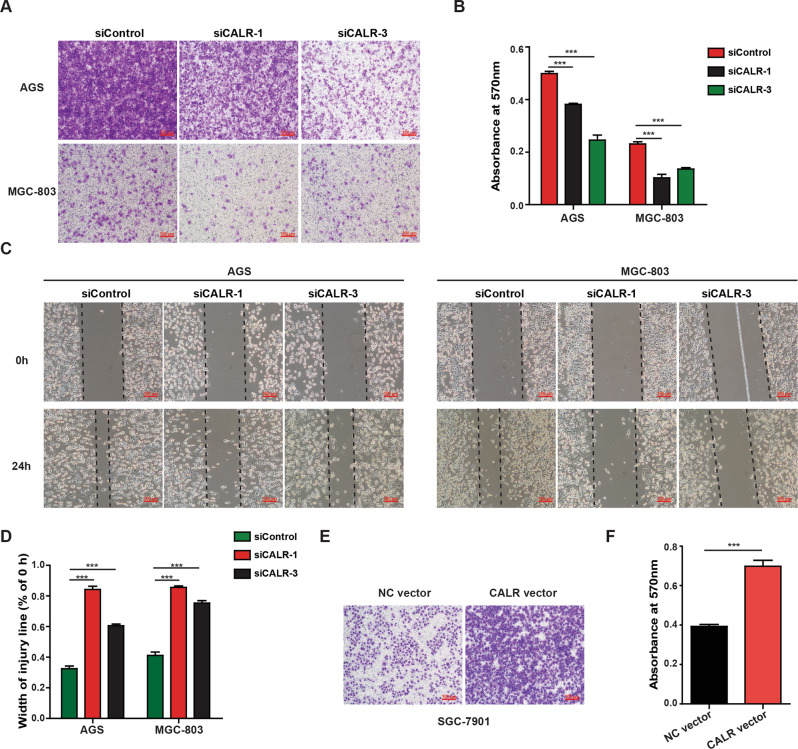
The effects of CALR on GC cells migration in vitro. **A**, **B** Transwell assays and (**C** and **D**) wound healing assays detecting migration abilities of GC cells transfected with CALR siRNAs or control in AGS and MGC-803 cells (scale bar = 100 μm). Wound healing was quantified by measurement of the average linear speed of movement of the wound edges. **E**, **F** Representative photomicrographs of transwell assays results for SGC-7901 cells infected with NC vector or CALR vector (scale bar = 100 μm). All of the experiments were performed three times. Data are presented as mean ± SEM. *P < 0.05, **P < 0.01, ***P < 0.001.

Our clinical findings revealed that CALR expression was correlated with the depth of invasion and lymph node metastasis in GC specimens, implying that CALR might involve in the invasion and metastasis of GC. Based on the results shown in Fig. 1G, H, we chose GC cell lines AGS and MGC-803 for CALR inhibition assays and GC cell line SGC-7901 for establishing CALR stable overexpression model to explore the effects of CALR on GC cell migration; the transfection and infection efficiencies of CALR mRNA and protein level were shown in Supplementary Fig. 2A–E. As shown in Fig. 2A–D, compared with control groups, knockdown of CALR markedly inhibited the migratory capabilities of AGS and MGC-803, while an opposite result was observed in CLAR-overexpressing SGC-7901 cells (Fig. 2E, F). Together, these results indicate that the profiles of CALR influence the migration of GC cells in vitro.

### CALR promotes GC cell migration in vivo

**Fig. 3:**
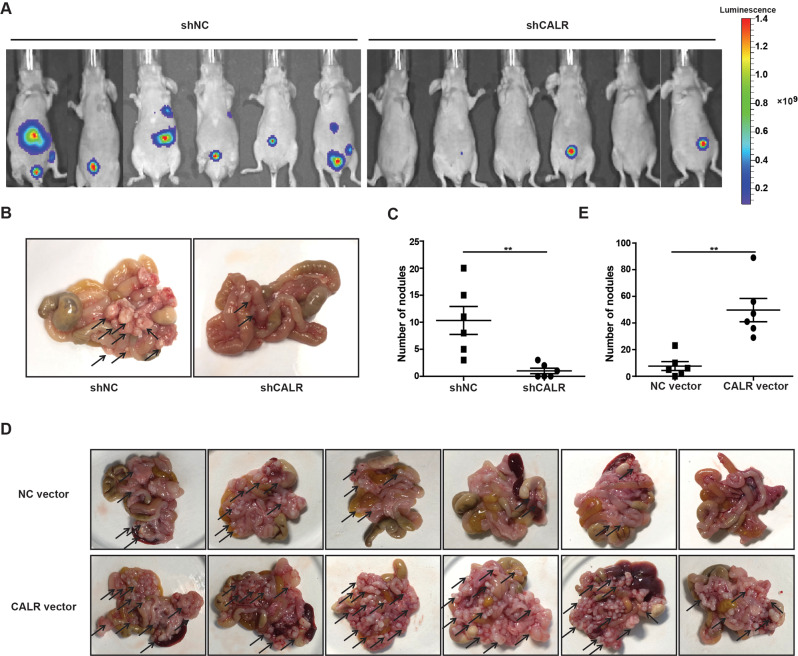
CALR regulated GC cells metastasis in vivo. Representative images of the GC abdominal spread model after respectively intraperitoneal injection with GC cells stably knocking down CALR/NC vector (**A** and **B**, *n* = 6 vs 6) and stably overexpressing CALR/NC vector (**D**, *n* = 6 vs 6). **C**, **E** Evaluation of the number of tumor nodules in mice abdominal cavities. Data are presented as mean ± SEM. *P < 0.05, **P < 0.01, ***P < 0.001.

Furthermore, to elucidate the role of CALR in GC development, we established the intraperitoneal metastasis models that stable CALR-knockdown MGC-803 cells, CALR overexpressing SGC-7901 cells, and their control cells were respectively injected into the intraperitoneal cavities of nude mice. After 28 days, the mice were sacrificed, and the results revealed that silencing CALR remarkably reduced the formation of metastatic nodules (Fig. 3A–C), whereas CALR overexpression remarkably elevated the number of metastatic nodules compared to the mice implanted with control cells. In summary, these results further support that CALR plays an important role in GC metastasis.

### CALR promotes migration metastasis of GC cells in association with induction of EMT

**Fig. 4:**
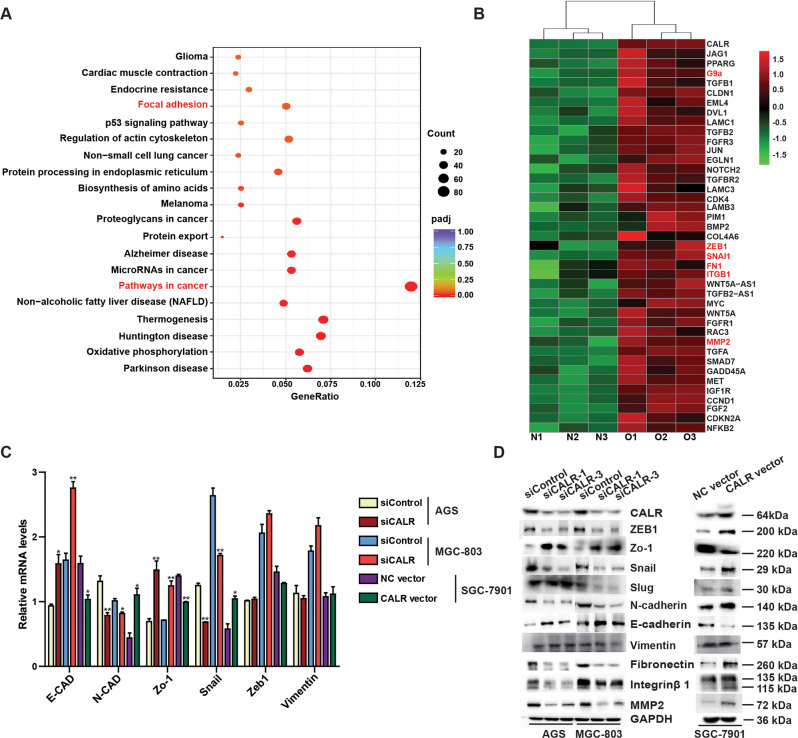
CALR promotes GC cells migration in vitro and metastasis in vivo in association with EMT. **A**, **B** KEGG dot analysis of changed genes involved pathways in CALR overexpressing model. **C** RT-qPCR and (**D**) western blotting assays verified the results of validated EMT related markers screening by transcriptome sequencing. Data are presented as mean ± SEM. *P < 0.05, **P < 0.01, ***P < 0.001.

To elaborate the mechanism by which the CALR induced GC cells migration, we conducted transcriptome sequencing of CALR stable overexpression model and its control cells. A total of 1522 changed genes (|log2 FoldChange | >0 and padj <0.05) were identified and included 721 upregulated genes and 801 downregulated genes (Supplementary Fig. 3A). KEGG Pathway Analysis revealed that overexpression of CALR remarkably affected cellular focal adhesion and pathways in cancer (Fig. 4A). More importantly, we observed that overexpression of CALR markedly promoted upregulation of EMT markers (Snail and ZEB1) and cell adhesion related molecules (MMP2, Fibronectin and integrin β1) (Fig. 4B). Besides, we still found the expression of CALR was positively related to Snail and negatively Zo-1 by analysis of TCGA data (Supplementary Fig. 3B, C). Increased cell migration is also a functional consequence of EMT [20], so we next examined changes in EMT-related markers by RT-qPCR and Western blot. Indeed, we observed that the inhibition of CALR dramatically decreased the mRNA and protein levels of N-cadherin and Snail and increased E-cadherin and ZO-1 mRNA and protein levels. The reverse results at mRNA and protein levels were observed by overexpression of CALR. The change of ZEB1 protein level was significant, while mRNA level had no difference when knocking down or overexpressing CALR in GC cells. In addition, there were no differences for other EMT markers (Vimentin and Slug) (Fig. 3C, D). Furthermore, according to the results of transcriptome sequencing, we also examined MMP2, Fibronectin, and integrin β1 protein expression, which played important roles in the adhesion of cells [21]. As expected, knockdown of CALR led to inhibition of MMP2, Fibronectin and integrin β1, while CALR overexpression conversely. Taken together, these results suggest that CALR enhances GC metastasis mainly by inducing EMT process and affecting cell adhesion.

### CALR induced EMT requires G9a-mediated H3K9 methylation

**Fig. 5:**
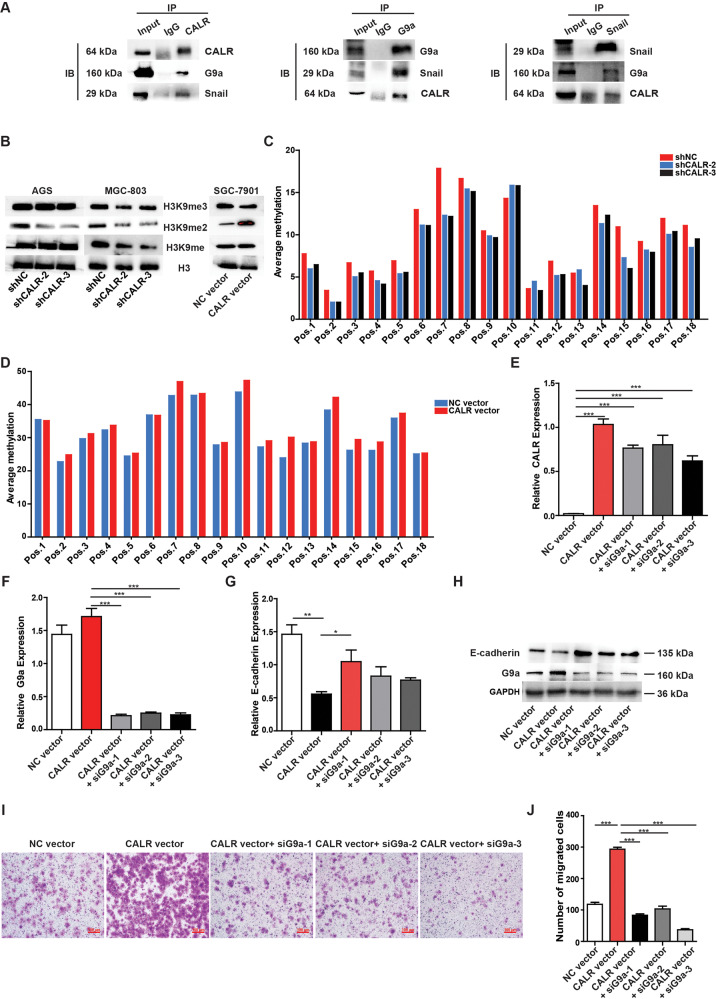
CALR induces EMT progression by repressing E-cadherin expression mediated by G9a. **A** The interactions among CALR, G9a and Snail were confirmed by immunoprecipitation. IgG was used as a negative control. **B** Western blot analysis of methyl-histone H3 (Lys9) in CALR overexpressing and knocking down models. **C**, **D** Methylation position analysis of E-cadherin promoter in CALR overexpressing and knocking down models via pyrosequencing. **E**–**G** RT-qPCR and (**H**) WB analysis the mRNA and protein level of CALR, G9a and E-cadherin in CALR overexpression models transfected with G9a siRNA or siControl. **I**, **J** Representative images of G9a siRNA counteracting the acceleration of CALR on GC cells migration in CALR overexpressing models.

As the results above showed, CALR mediated EMT process in GC cells, while the implying mechanism remained unclear. Therefore, proteins that could bind to CALR were examined by IP and MS assays. The list of all potential target proteins for interacting with CALR was provided in Supplementary Table 1. Intriguingly, we noticed that CALR could interact with G9a, which was a euchromatin-associated methyltransferase responsible for mono- and dimethylation of H3K9, and was also recruited to the E-cadherin promoter by Snail and mediated promoter DNA methylation of E-cadherin and the suppression of E-cadherin expression [22]. To validate the interaction of CALR with G9a, we conducted IP assays and immunoprecipitated endogenous CALR in GC cells. In line with this finding of MS analysis, G9a was pulled down by CALR antibody. Besides, CALR was also combined with Snail (Fig. 5A). Similar results were obtained when we used G9a or Snail antibody to immunoprecipitate endogenous CALR (Fig. 5A). Because the interaction between G9a and Snail played a critical role in inducing H3K9me2 and resulting in the DNA methylation of the E-cadherin promoter, we examined methylation levels of H3K9 and E-cadherin promoter by Western Blot assay, DNA Methylation Experiment, and Pyrosequencing. We found that the levels of H3K9me2 and DNA methylation on the E-cadherin promoter were higher in CALR overexpressed SGC-7901 cell lines, while the induction was repressed in the absence of CALR in AGS and MGC-803 cell lines (Fig. 5B–D). To further verify that loss of E-cadherin expression was associated with elevated G9a in GC cells, we blocked G9a expression in CALR overexpressed SGC-7901 cell lines and the efficiency of blocking was shown in (Fig. 5E, F). As expected, knockdown of G9a eliminated the repressed effect of CALR overexpression on E-cadherin expression (Fig. 5G, H). Functionally, consistent result was obtained by transwell assays that knockdown of G9a counteracted acceleration of CALR overexpression on GC cell migration (Fig. 5I, J). These data reveal a cooperative role between CALR and G9a in the suppression of E-cadherin expression and induction of GC metastasis.

## Discussion

Over the past years, numerous studies have demonstrated that CALR participated in various cancer cell invasion and migration and was related to patients’ unfavorable clinical prognosis [23–26]. In GC, researchers found that CALR overexpression enhanced angiogenesis and was associated with microvessel density, metastasis and survival via a independent cohort study of little GC patients [13, 27]. However, the mechanism and function of CALR inducing GC metastasis are obscure. Only Po-Chu Lee et al. [28] found that CALR bound with VEGF-A to stabilize VEGF-A mRNA, thereby promoting the angiogenesis and progression of gastric cancer. MiR-637 could aggravate endoplasmic reticulum stress-induced apoptosis by repressing CALR [29]. In this study, our results indicated that CALR overexpression was indeed relevant to depth of invasion, lymph node metastasis, prognosis, and recurrence in GC specimens via large data and our collected fresh GC samples analysis, which was in concordance with previous studies. More importantly, our results also hinted that serum CALR might serve as a potential biomarker for GC diagnosis. In addition, we established CALR stable overexpression or repression model in GC cells via lentiviral vector and demonstrated that CALR was a key mediator of GC cells migration by cell migration assays and animal assays.

To clarify the mechanism that CALR induced GC metastasis, we performed transcriptome sequencing. Intrestingly, the data showed that the overexpression of CALR dramatically elevated EMT-related markers (ZEB1 and Snail) levels and cellular adhesive molecular (Fibronectin, integrin β1and MMP2) expression. Similar results were obtained by WB assays and big data analysis, which also indicated CALR expression was relative to EMT markers (E-cadherin, ZO-1, and N-cadherin) expression. A cancer-related EMT usually drives early steps of metastatic cascade via transforming epithelial tumor cells into invasive metastatic cancer cells [30, 31]. A previous study by Weiwei Sheng et al. showed that CALR promoted EMT in pancreatic cancer via Integrin/EGFR-ERK/MAPK signaling pathway and mediating Ca^2+^ dependent acute and chronic endoplasmic reticulum stress [17, 32]. Other research revealed that CALR regulated EMT through modulating Smad signaling and calcium signaling in lung cancer cells [33] and via TGF-β/Smad3/NRP1 pathway in nasopharyngeal carcinoma cells [34]. However, the role of CALR inducing the EMT process in GC is unknown. In this context, with the help of transcriptome sequencing, MS and IP assays, we discovered that CALR could form complexes with G9a and Snail. Although Snail is highly expressed in GC for directly repressing E-cadherin expression [35], the sequential event and other molecular mechanisms leading to DNA methylation at the E-cadherin promoter in GC remain not well characterized [36]. G9a encoded by EHMT2 (euchromatic histone lysine N-methyltransferase 2) is a mammalian histone methyltransferase responsible for histone H3-lysine 9 methylation (H3K9) that contributes to the epigenetic silencing of tumor suppressor genes [37, 38]. Previous studies showed that G9a was upregulated in GC and controlled GC progression via the mTOR pathway [39] or upregulating ITGB3 in a SET domain-independent manner [40]. G9a interacts with Snail, DNA methyltransferases (DNMTs) and forms a complex, which is recruited to the E-cadherin promoter and results in E-cadherin promoter DNA methylation and the suppression of E-cadherin expression in claudin-low breast cancer [41]. However, whether CALR induces GC metastasis depending on G9a remains unclear. Here, our data indicated that CALR expression affected H3K9 dimethylation (H3K9me2) and methylation of E-cadherin promoter by cooperating with Snail and G9a. CALR expression was positively correlated with G9a and Snail. Knockdown of G9a in CALR overexpression model remarkably abrogated the aggravated effect of CALR overexpression on GC cells migration and suppression of E-cadherin mRNA and protein levels. This is the first study to reveal the important role of G9a-Snail complex in the activation of the CALR-mediated EMT process and GC metastasis.

Besides, our data also showed CALR could regulate cellular adhesive molecular (Fibronectin, integrin β1and MMP2) expression. Cell attachment plays an important role in cancer cells migration. Previous studies illustrated that many molecules induced GC metastasis by activating integrin signaling [15, 42–45]. Matrix metalloprotease-2 (MMP-2) plays a pivotal role in cancer invasion and metastasis. Min Wu et al. [46] found that CALR regulated MMP2 expression via the PI3 pathway. CALR is directly involved in anti-alpha3 integrin antibody-mediated secretion and activation of MMP2 in rhabdomyosarcoma cells [47]. Our results also demonstrate for the first time that CALR is involved in the regulation of Fibronectin, integrin β1and MMP2 expression in GC, which provides a crucial evidence for CALR mediated GC metastasis.

**Fig. 6:**
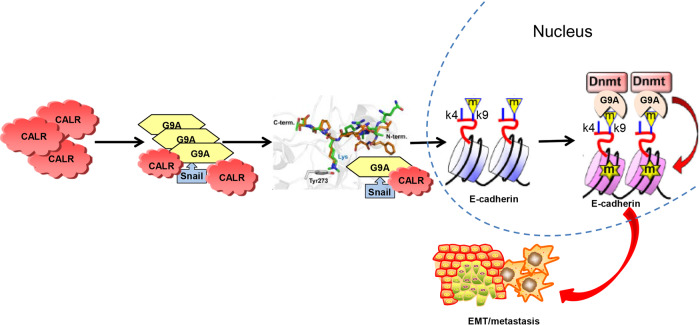
A schematic model depicting to illustrate the interaction of Snail with G9a and DNMTs leading to E-cadherin promoter methylation and EMT induction. G9a-mediated H3K9me2 can provide a docking site for the Dnmt complex, which results in DNA methylation at the E-cadherin promoter.

In summary, as displayed in the Fig. 6 schematic, our study demonstrated the expression levels of CALR and regulatory effect of CALR on GC cell migration in vitro and vivo and provided several insights into the regulatory effect of CALR on the methylation status of the E-cadherin promoter in GC, which provided promising therapeutic approaches in the treatment of metastatic GC with high CALR expression. Besides, CALR is a secreted protein, and our study shows it significantly elevated in GC patients’ serum. Therefore, the diagnostic values of the CALR protein level in the blood of GC patients areworth further investigation. In addition, CALR is also found in the nucleus, implying that it may have a role in transcriptional activities and gene expression regulation [5]. Research showed that CALR could be important in gene transcription by interacting with the DNA-binding domain of the glucocorticoid receptor and the synthetic peptide KLGFFKR, and regulating the glucocorticoid receptor and perhaps other members of the superfamily of nuclear receptors [48]. CALR also upregulated neuropilin-1 expression via STAT5A in esophageal cancer cells [49]. Therefore, how CALR is to regulate Snail and G9a expression in GC which deserves further study.

## Materials and methods

### Human specimens

All GC and paired adjacent nontumor tissues (at least 5 cm away from tumor edge) and serum samples in this study were collected from Southwest Hospital of Army Medical University (Chongqing, China). These patients were all confirmed by histopathological analysis and did not undergo chemotherapy, radiotherapy or immunotherapy at the time when tissues or serums samples were collected. Informed consents were obtained from every participants. This research abided by Helsinki Declaration and was performed with the permission of the Ethics Committee of Army Medical University.

### Cell culture

GC cell lines (AGS, MGC-803, XN0422, BGC-823, SGC-7901, and GES-1) were purchased from ATCC (Manassas, VA, USA) and were cultured as previously described [50, 51].

### Immunohistochemistry (IHC)

IHC staining and scoring were performed as previously described [51, 52]. Differently, the CALR antibody (Sigma-Aldrich, USA) diluted at 1:2000 was used for the primary reaction in this study.

### Lentiviral vector construction and infection

Human CALR overexpressing and negative control (NC) lentiviral vectors were purchased from GenePharma (Shanghai, China). SGC-7901 cells were infected with LV18-Puro-CALR lentiviral vector or LV18-Puro-NC vector according to the manufacturer’s protocol. Human CALR knocking down and NC lentiviral vector were purchased from Hanbio (Shanghai, China). MGC-803 and AGS cells were infected with HBLV-Luc-Puro-CALR lentiviral vectors or HBLV-Luc-Puro-NC vectors according to the manufacturer’s protocol. Then stable cell lines were selected with puromycin (1 μg/mL) for 2 weeks.

### Oligonucleotides and transfection

CALR, G9a siRNAs, and control siRNAs were synthesized by RIBOBIO (Guangzhou, China). Lipofectamine 2000 (Invitrogen, Carlsbad, CA) was used for all transfections in this study according to the manufacturer’s instructions.

### RNA isolation and quantitative real-time PCR (RT-qPCR)

Total RNA of GC cells and tissues was extracted via RNAiso reagent (Takara, Dalian, China). PrimeScript™ RT reagent Kit (TaKaRa, Dalian, China) was used for reverse transcription RT-qPCR reactions were conducted by SYBR^®^ Green Real-time PCR Master Mix (TOYOBO, Japan). The primers for GAPDH, Zo-1, Snail, ZEB1, Vimentin, E-cadherin and N-cadherin referred to Yuan Jihang’s [53]. The primers for CALR and G9a were as follows: CALR, (F) 5′-CAGACTCCAAGCCTGAGGAC-3′ and (R) 5′-CCTCCTCTTTGCGTTTCTTG -3′; G9a, (F) 5′-AGGCACCCAAGATTGACC-3′, (R) 5′-GTCTCCCGCTTGAGGATG-3′. All RT-qPCR reactions were performed in triplicate using a Bio-Rad CFX96 real-time PCR system. The gene expression data were normalized to GAPDH. The results were calculated in accordance with the 2^−ΔΔCT^ method.

### Transcriptome sequencing

A total amount of 3 μg RNA per sample was used as input material for the RNA sample preparations. Sequencing libraries, transcriptome sequencing, and computational analyses were fulfilled by Novogene (Beijing, China).

### Cell migration assays and wound healing assay

GC cells migration ability was evaluated by cell migration assays and wound healing assay whose detailed procedures were described previously [54].

### Western blot assay

CALR (1:1000) and Fibronectin (1:1000) polyclonal antibody were obtained from Abcam (Shanghai, China); GAPDH, E-cadherin, N-cadherin, G9a, ZEB1, ZO-1, Snail, Slug, Vimentin, Integrin β1, MMP2 antibodies and methyl-histone H3 (Lys9) antibody sampler kit (1:1000) were purchased from Cell Signaling Technology (Shanghai, China); GAPDH served as an internal reference. Anti-mouse IgG or anti-rabbit horseradish peroxidase (HRP)-conjugated was used as secondary antibody (1:10000; ZhongShan, Beijing, China). Signals were detected by using the SuperSignal West Dura Extended Duration Substrate Kit (Thermo Scientific, Shanghai, China) and the results were analyzed by Image J software (Bio-Rad, Hercules, USA).

### Immunoprecipitation and MS analysis

1 mg protein extracted from AGS cells was immunoprecipitated (IP) overnight with anti-CALR antibody (Abcam, Shanghai, China), anti-Snail antibody and anti-G9a antibody (Cell Signaling Technology, USA), or rabbit or normal mouse IgG (Beyotime, Jiangsu, China) at 4 °C and then with protein A + G agarose beads (Beyotime, Jiangsu, China) for at least 2 h. After IP, the potential proteins binding with CALR were analyzed by MS analysis (SANGON BIOTECH, Shanghai, China). To verify the results of MS analysis, the beads were then boiled in 30 μL of 1% SDS loading buffer for western blotting with Snail (Cell Signaling Technology, USA), G9a (Proteintech, USA), or CALR antibodies (Sigma-Aldrich, USA).

### Pyrosequencing

Pyrosequencing was performed with the help of Sinotech Genomics (Shanghai, China). Briefly, DNA was isolated from stably overexpressing or repressing CALR GC cells or their controls. Then 300–1000 ng DNA of each sample was used to bisulfite converted using EZ DNA Methylation Kits(Zymo Research, USA), then converted products were put into PCR amplification and Machine sequencing by Sinotech Genomics (Shanghai, China). PCR primers and Pyrosequencing primers were shown in Supplementary Table 2. Finally, The Pyro Q-CPG software of the pyrosequencing device was used to automatically analyze the methylation status of each site. Analysis of the difference between the target sequence and the actual read sequence information.

### Intraperitoneal dissemination assay

Intraperitoneal dissemination experiments were conducted as previously described [51]. 1 × 10^6^ stably overexpressing CALR SGC-7901 cells or repressing CALR MGC-803 cells or their controls were respectively injected into the abdominal cavity of female BABL/c nude mice (6–8 weeks old, *n* = 6 vs 6), which were approved by the Institutional Animal Care and Use Committee of Army Medical University. Four weeks later, the tumor metastasis in the abdominal cavity of the mice was observed by in vivo imaging system and counting the tumor nodules after the mice were sacrificed.

### Statistical analyses

Statistical differences were analyzed via GraphPad Prism 5.0 Software (La Jolla, CA, USA) using paired t-tests, Two-tailed Student’s t-tests, and One-way ANOVA as appropriate. The relationships between CALR and Snail/ZO-1/G9a expression levels were analyzed by Pearson’s correlation. Sensitivity and specificity of serum biomarkers were assessed using ROC curve and AUC. The results are presented as mean ± SEM. *P* ≤ 0.05 was considered statistical significance.

## Supplementary information

supplementary materials

supplementary table 1

supplementary table 2

Supplementary Figure1

Supplementary Figure2

Supplementary Figure3

## Data Availability

The data used and analyzed during the current study are available from the corresponding author on reasonable request.
