# Supplementary material for: Calreticulin enhances gastric cancer metastasis by dimethylating H3K9 in the E-cadherin promoter region mediating by G9a
Source: Oncogenesis. 2022 May 31;11(1):29. doi: 10.1038/s41389-022-00405-7 (PMC9156786; doi:10.1038/s41389-022-00405-7)
Supplement: Supplementary file 1 — supplementary materials [file 41389_2022_405_MOESM1_ESM.docx]

**Supplementary Materials and Methods**

**Methods**

**ELISA assay**

RayBio® Human Calreticulin ELISA Kit was obtained from RayBiotech (USA). Assay procedures were strictly according to manufacturers. Briefly, first, prepare and bring all reagents, samples and standards as instructed to room temperature. And then, add 100 μl standard or sample to each well for incubating 2.5 hours at room temperature. Next add 100 μl prepared biotin antibody to each well for incubating 1 hour at room temperature. Add 100 μl prepared Streptavidin solution for incubating 45 minutes at room temperature. Finally, add 100 μl TMB One-Step Substrate Reagent to each well for incubating 30 minutes at room temperature and add 50 μl Stop Solution to each well and read at 450 nm immediately.

**Supplementary Table 1** Potential specific proteins binding with CALR in AGS cells by MS analysis.

**Supplementary Table 2** Pyrosequencing and PCR primers.

**Supplementary Fig. 1** CALR is upregulated in primary GC tumor tissues and correlates with GC clinicopathological features. (A) CALR is dramatically upregulated in GC samples from TCGA dataset. (B-E) The correlation analysis between CALR expression and clinicopathological features of GC patients from the Oncomine dataset. CALR exhibited significantly higher expression in GC patients with advanced stage disease (B), metastasis (C) , postoperative recurrence (D) and involvement of lymphatic vessels (E). The AUCs of serum CALR and traditional biomarkers (CA19-9 and CEA) in distinguishing early stages GC patients from healthy controls are shown (F). **P* < 0.05, ***P* < 0.01, ****P* < 0.001.

**Supplementary Fig. 2** The effectiveness of knocked down or overexpressed CALR in GC cells. **(A)** RT-qPCR and **(D)** WB analysis of CALR mRNA expression or protein expression in AGS and MGC-803 cells transfected with siControl or CALR siRNA. siControl is the negative control. **(B and C)** RT-qPCR and **(E)** WB analysis of CALR mRNA expression or protein expression in GC cells infected with the CALR-overexpressing or CALR-knocking down lentivirus. In addition, the scramble vector was used as the negative control. The assays were repeated in duplicate. **P* < 0.05, ***P* < 0.01, ****P* < 0.001.

**Supplementary Fig. 3** (A) the total result of transcriptome sequencing for CALR stable overexpression model and its control cells. (B-D) the relationship analysis between CALR with EMT related molecules (Snail/ZO-1/G9a) from TCGA dataset.
