# Supplementary material for: Calreticulin enhances gastric cancer metastasis by dimethylating H3K9 in the E-cadherin promoter region mediating by G9a
Source: Oncogenesis. 2022 May 31;11(1):29. doi: 10.1038/s41389-022-00405-7 (PMC9156786; doi:10.1038/s41389-022-00405-7)
Supplement: Supplementary file 3 — supplementary table 2 [file 41389_2022_405_MOESM3_ESM.docx]

**PCR product(s)**

Forward primer: **ACTCCAGGCTAGAGGGTCACC**

Reverse primer: **CCGCAAGCTCACAGGTGCTTTGCAGTTCC**

1. **[Chromosome 16](http://www.ensembl.org/Homo_sapiens/contigview?chr=16&region=&start=68737112&end=68737329)** (len: 218)

68737112 **AC** **TCCAGGCTAG**

**AGGGTCACCG** **CG**TCTATG**CG** AGGC**CG**GGTG GG**CG**GGC**CG**T CAGCTC**CG**CC

CTGGGGAGGG GTC**CGCG**CTG CTGATTGGCT GTGGC**CG**GCA GGTGAACCCT

CAGCCAATCA G**CG**GTA**CG**GG GGG**CG**GTGCC TC**CG**GGGCTC ACCTGGCTGC

AGCCA**CG**CAC CCCCTCTCAG TGG**CG**T**CGGA** **ACTGCAAAGC** **ACCTGTGAGC**

**TTGCGG** 68737329

>16 dna:chromosome chromosome:GRCh38:16:68736912:68737529:1

CCACTGCACTCCAGCTTGGGTGAAAGAGTGAGACCCCATCTCCAAAA**CG**AACAAACAAAA

AATCCCAAAAAACAAAAGAACTCAGCCAAGTGTAAAAGCCCTTTCTGATCCCAGGTCTTA

GTGAGCCAC**CG**G**CG**GGGCTGGGATT**CG**AACCCAGTGGAATCAGAAC**CG**TGCAGGTCCCAT

AACCCACCTAGACCCTAGCAACTCCAGGCTAGAGGGTCAC**CGCG**TCTATG**CG**AGGC**CG**GG

TGGG**CG**GGC**CG**TCAGCTC**CG**CCCTGGGGAGGGGTC**CGCG**CTGCTGATTGGCTGTGGC**CG**G

CAGGTGAACCCTCAGCCAATCAG**CG**GTA**CG**GGGGG**CG**GTGCCTC**CG**GGGCTCACCTGGCT

GCAGCCA**CG**CACCCCCTCTCAGTGG**CG**T**CG**GAACTGCAAAGCACCTGTGAGCTTG**CG**GAA

GTCAGTTCAGACTCCAGCC**CG**CTCCAGCC**CG**GCC**CG**ACC**CG**AC**CG**CACC**CG**G**CG**CCTGCC

CT**CG**CT**CG**G**CG**TCCC**CG**GCCAGCC**ATG**GGCCCTTGGAGC**CG**CAGCCTCT**CG**G**CG**CTGCTG

CTGCTGCTGCAGGTACCC**CG**GATCCCCTGACTTG**CG**AGGGA**CG**CATT**CG**GGC**CG**CAAGCT

C**CGCG**CCCCAGCCCTG**CG**

**1S,Q48**


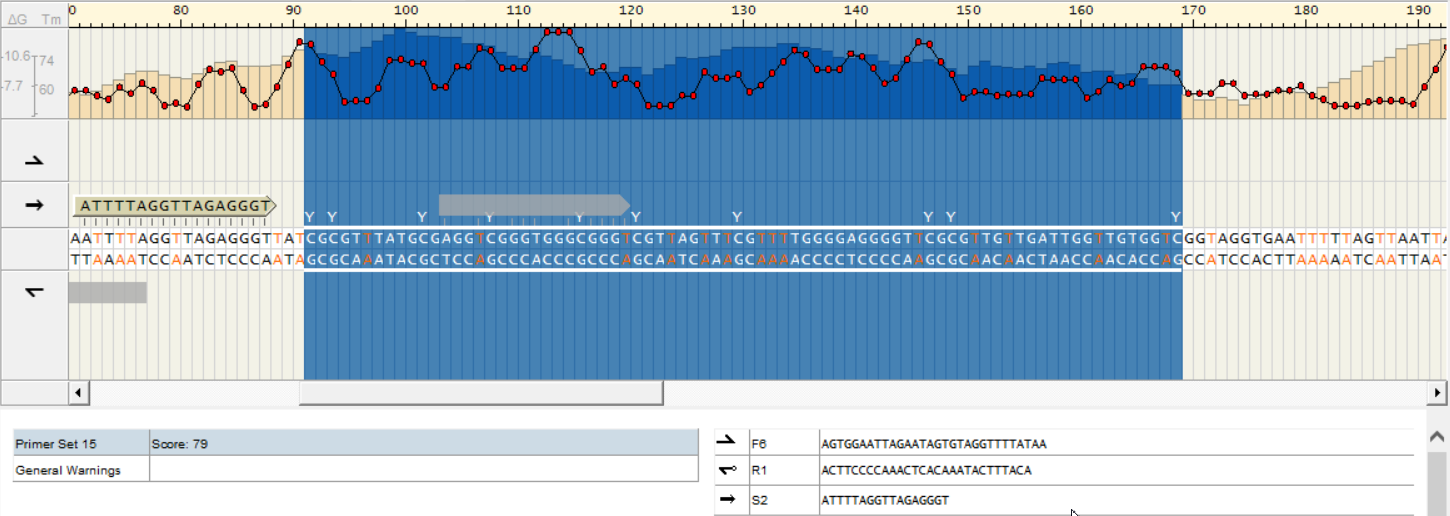


| \| Primer Set 15 \| Score: 79 \| \| --- \| --- \| \| General Warnings \| \|  \| \| --- \| \| | \| 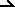 \| F6 \| AGTGGAATTAGAATAGTGTAGGTTTTATAA \| \| --- \| --- \| --- \| \| 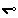 \| R1 \| ACTTCCCCAAACTCACAAATACTTTACA \| \| 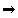 \| S2 \| ATTTTAGGTTAGAGGGT \| |
| --- | --- | --- | --- | --- | --- | --- | --- | --- | --- | --- | --- | --- | --- | --- | --- |

|  | PCR Product | Forward PCR Primer, F6 | Reverse PCR Primer, R1 | Sequencing Primer, S2 |
| --- | --- | --- | --- | --- |
| Length, nt | 270 | 30 | 28 | 17 |
| Position, 5'- 3' |  | 23 - 52 | 292 - 265 | 71 - 87 |
| Warnings | \| 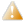 \| Deviation from optimal amplicon size \| \| --- \| --- \| | \|  \| \| --- \| | \|  \| \| --- \| | \|  \| \| --- \| |
| Tm, ºC |  | 57.2 | 57.8 | 44.2 |
| %GC | 34.8 | 26.7 | 35.7 | 35.3 |
| Sequence to Analyze | TATYGYGTTT ATGYGAGGTY GGGTGGGYGG GTYGTTAGTT TYGTTTTGGG GAGGGGTTYG YGTTGTTGAT TGGTTGTGGT YGGTAGGTGA ATTTTTAGTT AATTAG | | | |

**2S,Q48**


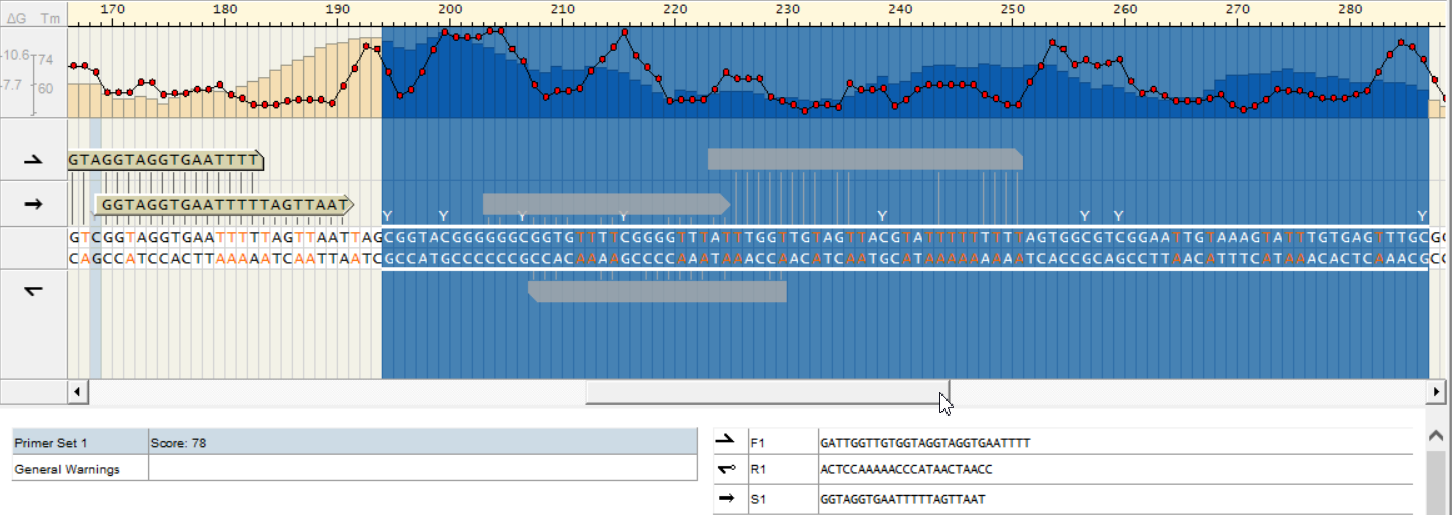


| \| Primer Set 1 \| Score: 78 \| \| --- \| --- \| \| General Warnings \| \|  \| \| --- \| \| | \| 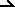 \| F1 \| GATTGGTTGTGGTAGGTAGGTGAATTTT \| \| --- \| --- \| --- \| \| 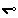 \| R1 \| ACTCCAAAAACCCATAACTAACC \| \| 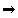 \| S1 \| GGTAGGTGAATTTTTAGTTAAT \| |
| --- | --- | --- | --- | --- | --- | --- | --- | --- | --- | --- | --- | --- | --- | --- | --- |

|  | PCR Product | Forward PCR Primer, F1 | Reverse PCR Primer, R1 | Sequencing Primer, S1 |
| --- | --- | --- | --- | --- |
| Length, nt | 235 | 28 | 23 | 22 |
| Position, 5'- 3' |  | 155 - 182 | 389 - 367 | 169 - 190 |
| Warnings | \|  \| \| --- \| | \|  \| \| --- \| | \|  \| \| --- \| | \|  \| \| --- \| |
| Tm, ºC |  | 62.7 | 59.8 | 44.9 |
| %GC | 31.9 | 39.3 | 39.1 | 27.3 |
| Sequence to Analyze | TAGYGGTAYG GGGGGYGGTG TTTTYGGGGT TTATTTGGTT GTAGTTAYGT ATTTTTTTTT AGTGGYGTYG GAATTGTAAA GTATTTGTGA GTTTGYGGAA GTTAGTTTAG ATTTTAGTT | | | |
