# Supplementary figures and images for: Calreticulin enhances gastric cancer metastasis by dimethylating H3K9 in the E-cadherin promoter region mediating by G9a
Source: Oncogenesis. 2022 May 31;11(1):29. doi: 10.1038/s41389-022-00405-7 (PMC9156786; doi:10.1038/s41389-022-00405-7)

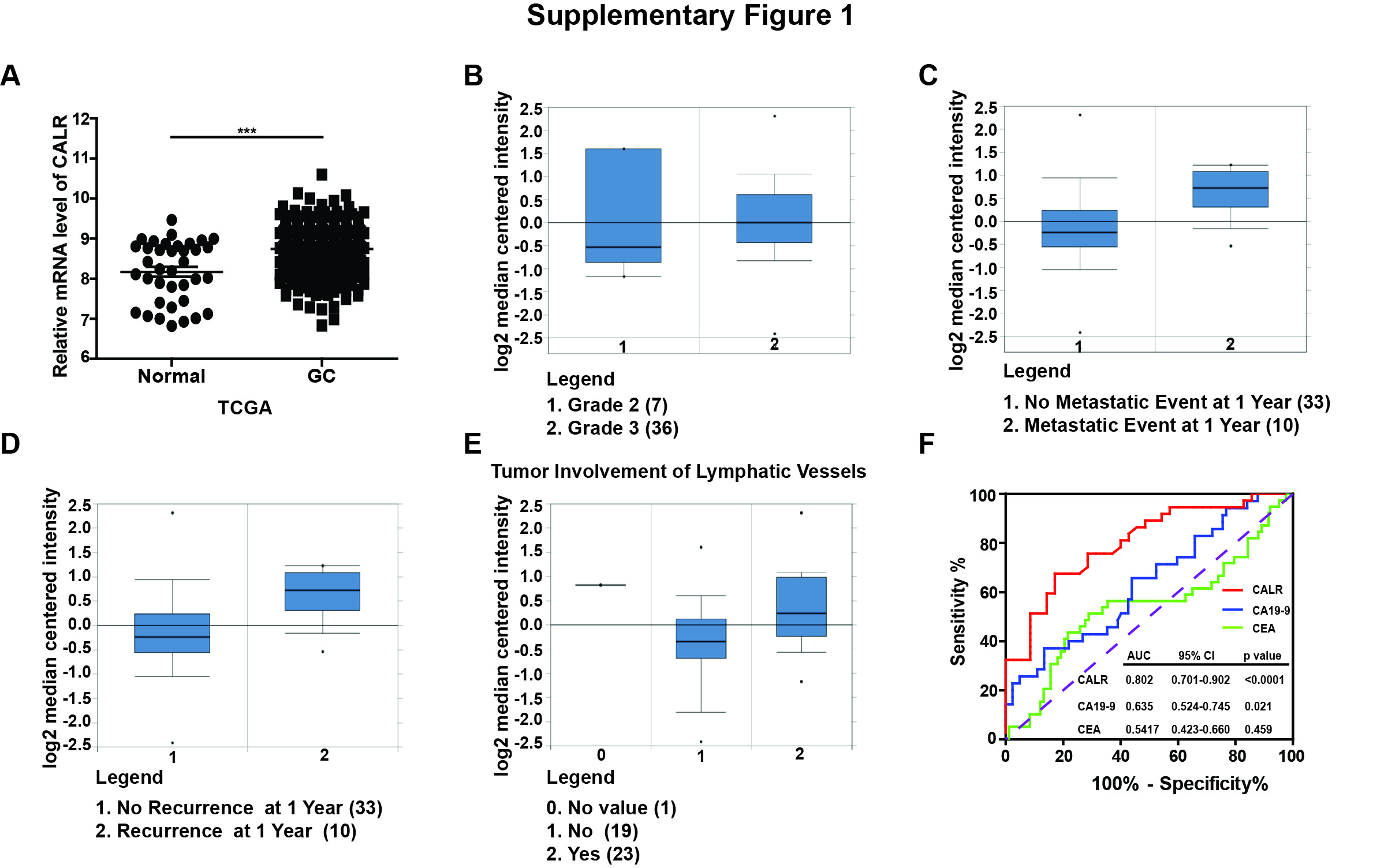

Supplement: Supplementary file 4 — Supplementary Figure1 [file 41389_2022_405_MOESM4_ESM.jpg]

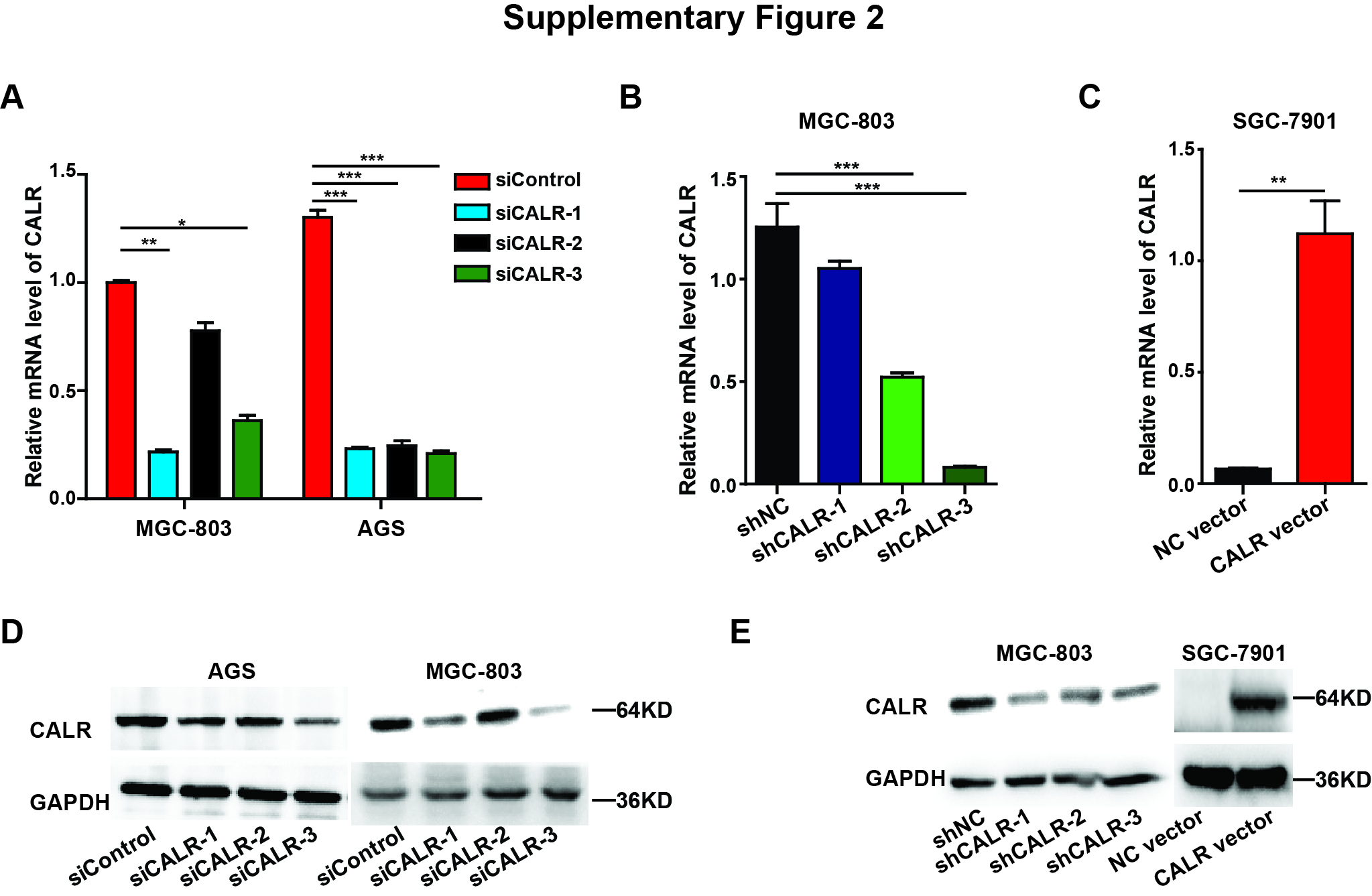

Supplement: Supplementary file 5 — Supplementary Figure2 [file 41389_2022_405_MOESM5_ESM.jpg]

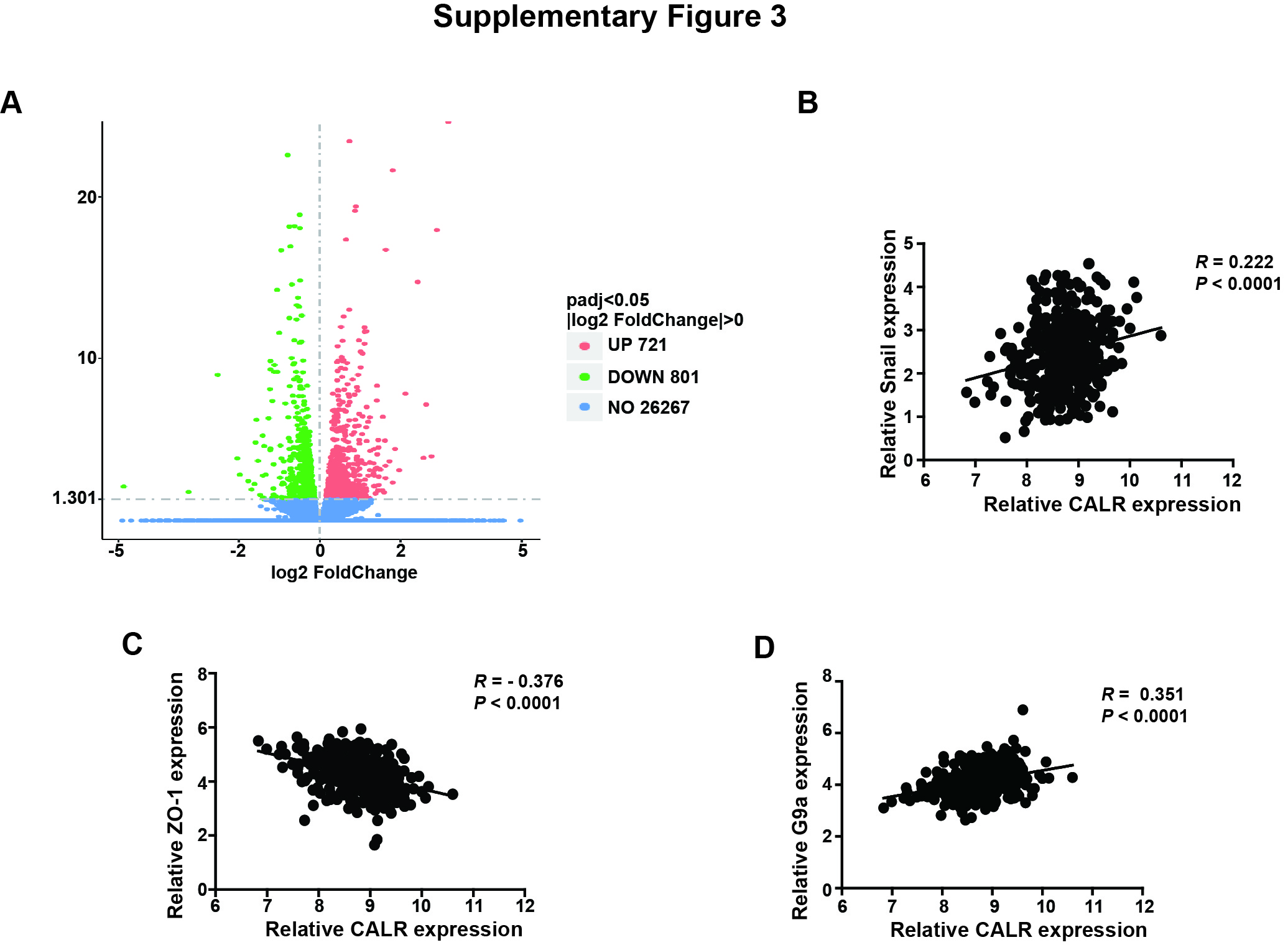

Supplement: Supplementary file 6 — Supplementary Figure3 [file 41389_2022_405_MOESM6_ESM.jpg]
